# The *C* *lostridium difficile* cell wall protein CwpV confers phase‐variable phage resistance

**DOI:** 10.1111/mmi.13121

**Published:** 2015-08-08

**Authors:** Ognjen Sekulovic, Maicol Ospina Bedoya, Amanda S. Fivian‐Hughes, Neil F. Fairweather, Louis‐Charles Fortier

**Affiliations:** ^1^ Département de microbiologie et d'infectiologieFaculté de médecine et des sciences de la santéUniversité de SherbrookeSherbrookeQuébecCanada; ^2^ Centre for Molecular Bacteriology and InfectionDepartment of Life SciencesImperial College LondonLondonUK; ^3^ Present address: Synthace Ltd.The London Bioscience Innovation Centre2 Royal College StreetNW1 0NHLondonUK

## Abstract

Bacteriophages are present in virtually all ecosystems, and bacteria have developed multiple antiphage strategies to counter their attacks. *C* *lostridium difficile* is an important pathogen causing severe intestinal infections in humans and animals. Here we show that the conserved cell‐surface protein CwpV provides antiphage protection in *C* *. difficile*. This protein, for which the expression is phase‐variable, is classified into five types, each differing in their repeat‐containing C‐terminal domain. When expressed constitutively from a plasmid or the chromosome of locked ‘ON’ cells of *C* *. difficile* R20291, CwpV conferred antiphage protection. Differences in the level of phage protection were observed depending on the phage morphological group, siphophages being the most sensitive with efficiency of plaquing (EOP) values of < 5 × 10^−7^ for phages ϕCD38‐2, ϕCD111 and ϕCD146. Protection against the myophages ϕMMP01 and ϕCD52 was weaker, with EOP values between 9.0 × 10^−3^ and 1.1 × 10^−1^. The C‐terminal domain of CwpV carries the antiphage activity and its deletion, or part of it, significantly reduced the antiphage protection. CwpV does not affect phage adsorption, but phage DNA replication is prevented, suggesting a mechanism reminiscent of superinfection exclusion systems normally encoded on prophages. CwpV thus represents a novel ubiquitous host‐encoded and phase‐variable antiphage system in *C* *. difficile*.

## Introduction

With an estimated 10^31^ particles in the biosphere, bacteriophages (phages) outnumber bacteria by a factor of at least 10. This means phages are present in virtually all ecosystems (Hatfull, 2008). Bacteria sometimes benefit from the incorporation into their genome of new prophages (i.e. integrated phages) that improve their fitness and/or virulence (Brussow *et al*., 2004; Fortier and Sekulovic, 2013). Nevertheless, bacteria have evolved multiple strategies to protect themselves from lytic phage attacks (Labrie *et al*., 2010).

These strategies aim at hampering various steps of the infection process, from phage adsorption to DNA injection, DNA replication and maturation, transcription and translation [for a review see (Labrie *et al*., 2010)]. For example, bacteria can modify or block receptors onto which phages adsorb to initiate the infection. Restriction‐modification (R‐M) systems are also widespread and cleave the incoming phage DNA (Pingoud *et al*., 2005). Abortive infection (Abi) systems are mechanisms of ‘innate immunity’ adopted by bacteria to limit phage propagation. This ‘altruistic suicide’ strategy aims at protecting the uninfected surrounding cells and keeping phage populations at a minimum, but the net result is death of the infected cells (Chopin *et al*., 2005). Clustered regularly interspaced short palindromic sequences (CRISPRs) represent an RNA‐based ‘adaptive immunity’ system protecting cells from phage infection and from the transfer of foreign DNA (Barrangou *et al*., 2007).

Superinfection exclusion (Sie) systems prevent phage infection by interfering at a very early step in the infection process. Contrary to other antiphage systems that are mostly encoded on plasmids or on the chromosome of the bacterial host, Sie are generally encoded by temperate phages. When a Sie‐encoding phage integrates into the chromosome of its host to initiate a lysogenic cycle, it expresses a protein that prevents lytic reinfection (or superinfection) by the same phage or related phages. The mode of action of Sie is blocking of phage DNA entry. Some Sie systems consist in membrane‐bound proteins that act as molecular decoys to the infection process. For example, Sie proteins are thought to prevent infecting phages from bridging with the cytoplasmic membrane or membrane protein(s) required for DNA injection, by interacting directly with a phage structural component. Alternatively, Sie proteins could possibly mask host‐encoded DNA injection proteins, thereby making them unavailable for phage infection (McGrath *et al*., 2002). Several Sie systems have been described in Gram‐negative bacteria, including the Imm and Sp membrane proteins encoded by coliphage T4, and the Sim and SieA systems encoded by many prophages from *Enterobacteriaceae* species (Labrie *et al*., 2010). Imm changes the conformation of the phage DNA injection site, thereby preventing DNA entry into the cytoplasm. Sp inhibits the lysozyme activity located at the tip of the T4 phage tail, which is required to drill a hole into the peptidoglycan wall to enable DNA injection (Lu and Henning, 1994).

In Gram‐positive bacteria, a few Sie systems have been identified, mainly in phages of *Lactococcus lactis* (McGrath *et al*., 2002; Mahony *et al*., 2008) and *Streptococcus thermophilus* (Sun *et al*., 2006; Bebeacua *et al*., 2013). They are all predicted or have been shown to be membrane‐associated proteins that function by blocking phage DNA injection. The prototype Sie system in *L. lactis* is Sie_2009_ encoded by the lactococcal phage Tuc2009. Although Tuc2009 is a member of the P335 phage species (Jarvis *et al*., 1991), Sie_2009_ confers resistance to a genetically distinct group of lactococcal phages, the 936 (McGrath *et al*., 2002). Additional genes encoding similar Sie systems have been identified in multiple lactococcal strains (McGrath *et al*., 2002; Mahony *et al*., 2008). In *S. thermophilus*, the temperate phage TP‐J34 encodes Ltp, a 142 amino acid lipoprotein conferring antiphage activity against similar *S. thermophilus* phages, but that also provides strong protection against a completely distinct group of *L. lactis* phages, in particular P008 (Mahony *et al*., 2006). Analysis of P008 mutants capable of overcoming the Ltp system revealed mutations in the tail tape measure protein (TMP). Crystal structure analysis of Ltp suggests that the antiphage activity results from the interaction between Ltp and the phage TMP while it is being ejected from the tail tube during infection, thereby preventing the formation of a channel through the bacterial membrane for passage of the viral DNA (Bebeacua *et al*., 2013).

*Clostridium difficile* is the main cause of antibiotic‐associated diarrhea in industrialized countries (Lo Vecchio and Zacur, 2012). Although many phages infecting *C. difficile* have been described (Hargreaves and Clokie, 2014; Sekulovic *et al*., 2014), no functional antiphage system has been functionally characterized in this species so far. Nevertheless, bioinformatics analyses revealed the presence of putative Abi systems in a few phage genomes, including AbiF in phage ϕC2 (ORF37) (Goh *et al*., 2007; Hargreaves *et al*., 2014) and an Abi‐like protein (CDR20291_1462) in the phi‐027 prophage present in all ribotype 027 isolates, including the R20291 (Stabler *et al*., 2009). In addition, CRISPRs and CRISPR‐associated (Cas) proteins are widely distributed in *C. difficile* genomes (Hargreaves *et al*., 2014), but although the system seems to be functional (Soutourina *et al*., 2013), an antiphage phenotype has never been demonstrated experimentally. Likewise, the CdiCD6I/M.CdiCD6I and CdiCD6II/M.CdiCD6II R‐M systems have been described in *C. difficile*, but their activity against phages has never been tested (Purdy *et al*., 2002).

CwpV is a conserved cell wall protein present in all *C. difficile* isolates and which is the largest member of the family of cell wall proteins (CWPs). Five different types of CwpV have been described to date, each differing in its C‐terminal domain. The characteristic feature of this domain is the presence of 4–9 tandem repeats of amino acids, each repeat comprising between 79 and 120 amino acids (Reynolds *et al*., 2011). CwpV is exported to the cell surface through a secA2‐dependent secretion system (Fagan and Fairweather, 2011). Once exported, auto‐processing of the protein at a specific cleavage site generates an N‐terminal fragment of ∼ 42 kDa and a C‐terminal fragment of variable size depending on the CwpV type. The two fragments then re‐associate through non‐covalent bonding into a heterodimer to generate the mature CwpV protein, which is anchored to the cell wall via three CWB2 cell wall–anchoring domains (Dembek *et al*., 2012; Willing *et al*., 2015). CwpV is a major constituent of the *C. difficile* cell wall, representing ∼ 13% of the total surface layer proteins (Reynolds *et al*., 2011). The biological function of CwpV is still unclear, although CwpV was shown to promote bacterial aggregation *in vitro*, suggesting a possible implication in gut colonization (Reynolds *et al*., 2011).

One of the characteristic features of CwpV is its phase‐variable expression, which is conserved among all CwpV types identified (Emerson *et al*., 2009). Only ∼ 5% of bacteria from a culture actively transcribe the *cwpV* gene. The site‐specific recombinase RecV catalyzes the recombination of a genetic switch located between the gene and the promoter, thereby turning ‘ON’ and ‘OFF’ the expression of *cwpV* (Emerson *et al*., 2009).

In this study, we provide experimental evidence showing that the conserved cell wall protein CwpV from *C. difficile* has antiphage activity. We show that cells turning the expression of *cwpV* ‘ON’ become resistant to infection by different phages, including members of the *Siphoviridae* and *Myoviridae* families. Our data strongly support a model in which CwpV prevents phage DNA from entering the cell, which is a mechanism reminiscent of superinfection exclusion systems encoded by other temperate phages.

## Results

### CwpV protects against phage infection

In a previous transcriptomic study with *C. difficile* R20291, we isolated a lysogen carrying the episomal ϕCD38‐2 prophage in which the *cwpV* gene (CDR20291_0440) was upregulated about 20 fold (Sekulovic and Fortier, 2015). This observation was intriguing, and although it is still unknown how ϕCD38‐2 interferes with *cwpV* expression, it raised interesting questions. The *cwpV* gene encodes a cell wall protein present in all *C. difficile* isolates analyzed to date. Although the function of the protein remains uncertain, experimental evidence suggests that it participates in bacterial aggregation, which could possibly contribute to colonization of the gut mucosa (Reynolds *et al*., 2011). Of note, *cwpV* expression is subject to phase variation, i.e. only a fraction of a bacterial population expresses the gene (Emerson *et al*., 2009). The biological reason for such phase variation is still unknown, and different hypotheses have been proposed. One of them is that CwpV could possibly participate in resistance to bacteriophage infection (Reynolds *et al*., 2011). Indeed, a number of phage receptors and phage resistance systems are subject to phase variation (Hoskisson and Smith, 2007; Seed *et al*., 2012). We therefore sought to verify if CwpV could protect against phage infection in *C. difficile*.

We used the R20291 strain, a ribotype 027 epidemic isolate that is susceptible to infection by three related temperate siphophages from our collection: ϕCD38‐2, ϕCD111 and ϕCD146 (Sekulovic *et al*., 2011; 2014). We also used the R20291_OFF_ strain, a R20291 mutant in which *cwpV* is not expressed (Sekulovic and Fortier, 2015) (Table 1). This mutant was created by inactivating the *recV* gene encoding the site‐specific recombinase RecV, using the ClosTron system (Heap *et al*., 2007; Reynolds *et al*., 2011). Inactivation of *recV* prevents recombination of the epigenetic switch controlling transcription of *cwpV* (Emerson *et al*., 2009), thereby allowing the isolation of clones in which the switch is locked in the ON or OFF configuration. The absence of *cwpV* expression in R20291_OFF_ was confirmed by RT‐qPCR, Western blot and immunofluorescence (Sekulovic and Fortier, 2015).

**Table 1:** List of bacterial strains, plasmids and phages used in this study

Strain, plasmid or phage	Characteristic or description	Reference or source
*C. difficile*		
R20291	Epidemic isolate, ribotype 027	Stabler *et al*. (2006)
R20291_OFF_	R20291 Cd1004‐144a::CT, *recV* mutant, *cwpV* genetic switch OFF	Sekulovic and Fortier (2015)
R20291_OFF_lysogen	R20291_OFF_ strain, carrying the ϕCD38‐2 prophage	This study
R20291_ON_	R20291 Cd1004‐144a::CT, *recV* mutant, *cwpV* genetic switch ON	This study
R20291_ON_lysogen	R20291_ON_ strain, carrying the ϕCD38‐2 prophage	This study
R20291_OFF_(*cwpV‐I*)	R20291_OFF_ containing pCBR080, *cwpV‐I* full length	This study
R20291_OFF_(*cwpV‐III*)	R20291_OFF_ containing pCBR106, *cwpV‐III* full length	This study
R20291_OFF_(*cwpV‐IV*)	R20291_OFF_ containing pCBR107, *cwpV‐IV* full length	This study
R20291_OFF_(*cwpV‐V*)	R20291_OFF_ containing pCBR109, *cwpV‐V* full length	This study
R20291_OFF_(*cwpV‐II*)	R20291_OFF_ containing pOS200, *cwpV‐II* full length	This study
R20291_OFF_(*cwpV‐II* N‐term)	R20291_OFF_ containing pOS201, *cwpV‐II* N‐term fragment	This study
R20291_OFF_(*cwpV‐II* 3reps)	R20291_OFF_ containing pOS202, *cwpV‐II* 3 repeats	This study
R20291_OFF_(*cwpV‐II* ΔSigP )	R20291_OFF_ containing pOS203, *cwpV‐II* ΔSignalP	This study
CD384	Human isolate	Sirard *et al*. (2011)
CD384(pRPF144E)	CD384 containing pRPF144E	This study
CD384(*cwpV‐I*)	CD384 containing pCBR080, *cwpV‐I* full length	This study
CD384(*cwpV‐III*)	CD384 containing pCBR106, *cwpV‐III* full length	This study
CD384(*cwpV‐V*)	CD384 containing pCBR109, *cwpV‐V* full length	This study
*Escherichia coli*		
CA434	HB101 carrying plasmid R702	Purdy *et al*. (2002)
Phage		
ϕCD38‐2	*Siphoviridae*	Sekulovic *et al*. (2011)
ϕCD111	*Siphoviridae*	Sekulovic *et al*. (2014)
ϕCD146	*Siphoviridae*	Sekulovic *et al*. (2014)
ϕMMP01	*Myoviridae*	Meessen‐Pinard *et al*. (2012)
ϕCD52	*Myoviridae*	Fortier and Moineau (2007)
Plasmid		
pRPF144	*Pcwp2‐gusA* cassette from pRPF137 subcloned into pMTL960	Fagan and Fairweather (2011)
pRPF144E	pRPF144 without *gusA* and with a unique BamHI site	This study
pCBR080	pMTL960 containing the full‐length *cwpV‐I* gene from strain 630 with C‐terminal streptavidin‐tag	Reynolds *et al*. (2011)
pCBR106	pMTL960 containing the full‐length *cwpV‐III* gene from strain CDKK167 with C‐terminal streptavidin‐tag	Reynolds *et al*. (2011)
pCBR107	pMTL960 containing the full‐length *cwpV‐IV* gene from strain M9 with C‐terminal streptavidin‐tag	Reynolds *et al*. (2011)
pCBR109	pMTL960 containing the full‐length *cwpV‐V* gene from strain AY1 with C‐terminal streptavidin‐tag	Reynolds *et al*. (2011)
pOS200	pRPF144 containing the full‐length *cwpV‐II* gene from strain R20291	This study
pOS201	pRPF144 containing the N‐terminal domain of the *cwpV‐II* gene from strain R20291	This study
pOS202	pRPF144 containing the N‐terminal domain + 3 proximal repeats of the *cwpV‐II* gene from strain R20291	This study
pOS203	pRPF144 containing the *cwpV‐II* gene with deleted signal peptide from strain R20291	This study

**Figure 1:**
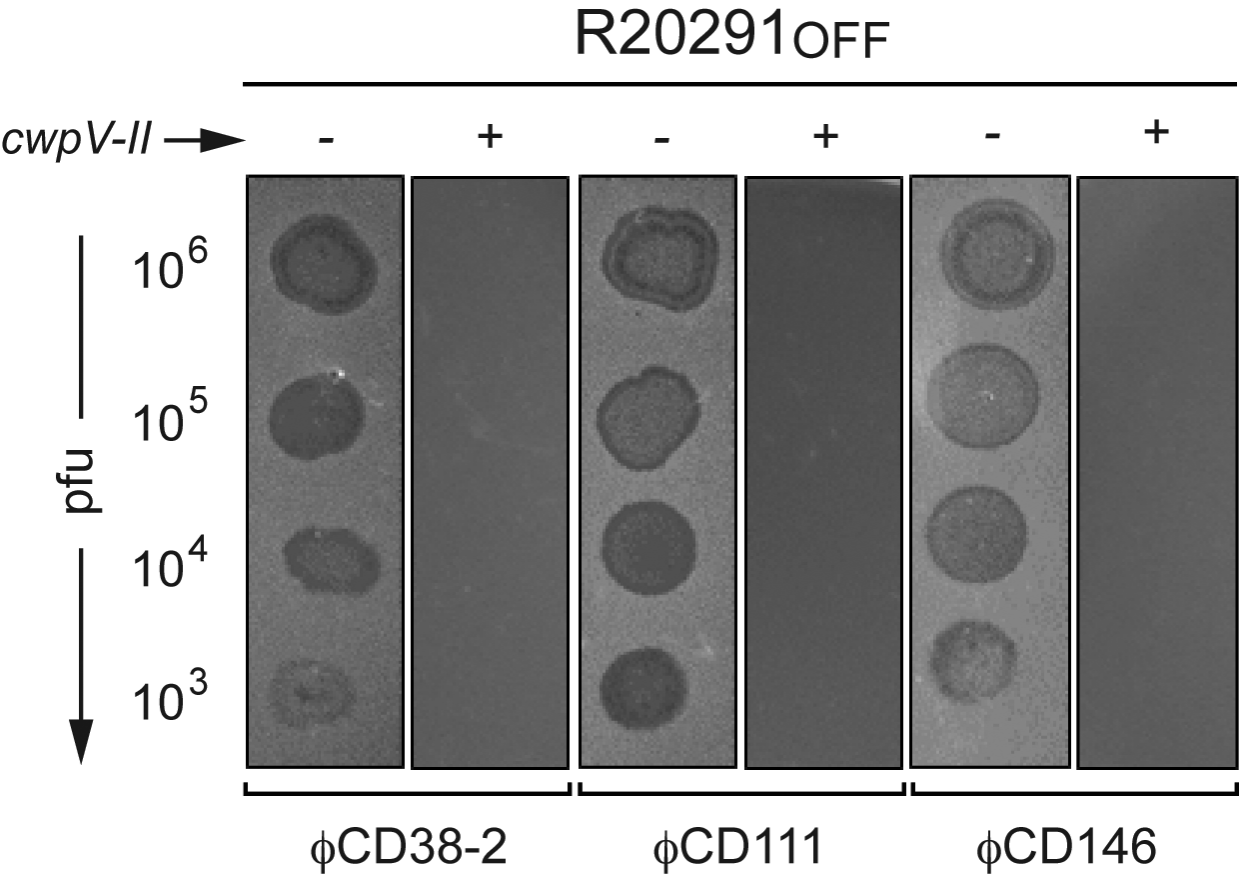
Susceptibility of *C* *. difficile* to phage infection in spot‐test assays. Bacterial lawns were prepared with the wild‐type R20291, or the R20291_OFF_ mutant strain, carrying (+) or not (−) the pOS200 plasmid expressing the *cwp* *V* type II. A 5 μl drop containing different titers of the phages ϕCD38‐2, ϕCD111 or ϕCD146 were then deposited on top of the lawns. Zones of clearing after incubation denote susceptibility to phage infection. The assay has been repeated at least three times and a representative result is shown.

**Figure 2:**
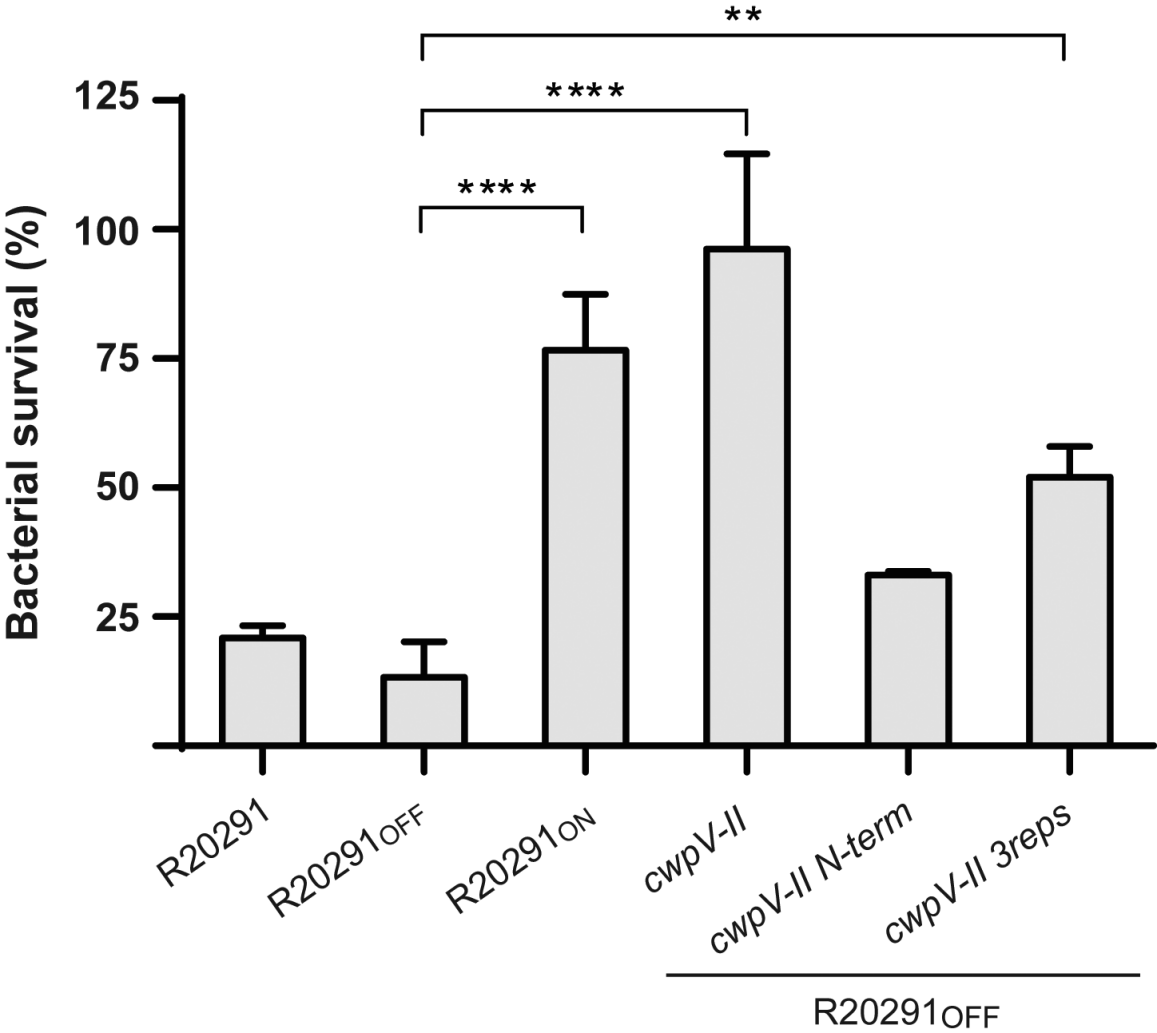
Bacterial survival following infection with phage ϕCD38‐2. Bacteria were infected with phage ϕCD38‐2 at a MOI of 1, and were subsequently plated after 15 minutes of incubation. The R20291_ON_ (locked ‘ON’) and R20291_OFF_ (locked ‘OFF’) strains were compared with the wild‐type R20291. Plasmids carrying either the full‐length *cwp* *V*‐II (pOS200), a truncated version with only the N‐terminal portion (pOS201) or a partially truncated version lacking the distal 5 repeats (pOS202) were also tested in R20291_OFF_. Colonies representing bacteria that survived the infection were counted and the result is expressed as a percentage of the ratio between infected and uninfected controls. Vertical bars represent means ± standard deviation (SD) of three independent biological replicates, which were each plated in technical triplicates. One‐way ANOVA comparisons were done with R20291_OFF_ as the reference condition (**P < 0.01; ****P < 0.0001).

We then cloned the R20291 type II *cwpV* gene (*cwpV‐II*) on the pRPF144 plasmid (Fagan and Fairweather, 2011), under the control of the constitutive promoter *P_cwp2_*, leading to pOS200 (Table 1). This plasmid was transferred by conjugation into the R20291_OFF_ mutant that does not express the chromosomal copy of *cwpV*. The presence of CwpV‐II at the bacterial surface was verified by Western blot and immunofluorescence on stationary‐phase cells (Figs. S1 and S2). Next, using a spot‐test infection assay, we assessed the impact of the overexpression of *cwpV‐II* on the susceptibility to infection by ϕCD38‐2, ϕCD111 and ϕCD146. While the R20291_OFF_ strain was fully susceptible to infection by all three phages, as it is the case for the wild type strain (Sekulovic *et al*., 2014) (not shown here), we observed a complete absence of infection in strain R20291_OFF_(*cwpV‐II*) overexpressing *cwpV‐II* (Fig. 1). In spot‐test assays, the relative multiplicity of infection (MOI) is very high at the point of phage deposition, especially with high titer dilutions. Therefore, we also assessed the influence of *cwp‐II* expression on susceptibility to phage infection in conditions where phages and bacteria were in a ratio of 1 to 1 (MOI = 1). We performed a cell survival assay in which we infected *C. difficile* in broth with ϕCD38‐2, followed by plating of the infected bacteria. As shown in Fig. 2, 96.2% ± 18.5% of the R20291_OFF_(*cwpV‐II*) bacteria survived the infection, whereas only 13.3% ± 6.8% of the R20291_OFF_ bacteria survived. As a comparison, we also infected the parental wild type R20291 strain, and 20.8% ± 2.5% of bacteria survived. These results further confirmed that overexpression of *cwpV‐II* confers phage resistance and that the complete absence of CwpV from the cell surface of the R20291_OFF_ strain increases susceptibility to phage infection.

Because the R20291_OFF_(*cwpV‐II*) strain overexpresses *cwpV* from an unrelated constitutive promoter on a plasmid, which does not reflect the natural condition, we constructed a strain in which *cwpV* was expressed from its own promoter on the chromosome. This was done by selecting *recV* mutants in which the genetic switch controlling the expression of *cwpV* was locked in the ‘ON’ configuration, i.e. strain R20291_ON_ (Table 1). The constitutive expression of *cwpV* in all cells of the R20291_ON_ strain was confirmed by immunofluorescence (Fig. S1). A strong protection against ϕCD38‐2 infection was observed in bacterial survival assays performed with the R20291_ON_ strain, with a survival rate of 76.7% ± 10.8% (Fig. 2). This suggests that under normal conditions, when an individual cell expresses *cwpV‐II*, phage infection is inhibited efficiently in that cell.

The presence of surviving R20291_OFF_ cells after phage exposure suggested that some cells were not infected, or some of them became lysogenic. Once a lysogen is formed, it becomes resistant to further killing by the same phage. We used an MOI of 1 in our survival assay to minimize lysogeny, which is promoted at higher MOI (Oppenheim *et al*., 2005). However, at an MOI of 1, we expected that some cells were not infected, and we could not rule out the possibility that lysogens also formed during the process. We therefore determined the proportion of lysogens among surviving cells following infection with ϕCD38‐2. We randomly picked 30 colonies from each infection experiment and analyzed them by PCR for the presence of ϕCD38‐2 using specific primers (LCF 312 and LCF 313; Table S1). No lysogens could be detected in R20291_OFF_(*cwpV‐II*), but 3/30 (10%) of survivors from R20291 and 6/30 (20%) from R20291_OFF_ were positive for ϕCD38‐2, confirming that lysogens were formed during the assay. However, most of the non‐lysogenic colonies were likely non‐infected cells. It is also noteworthy to mention that no lysogens could be detected following infection of the R20291_ON_ strain.

### CwpV protects against *S* *iphoviridae* phage infection

**Figure 3:**
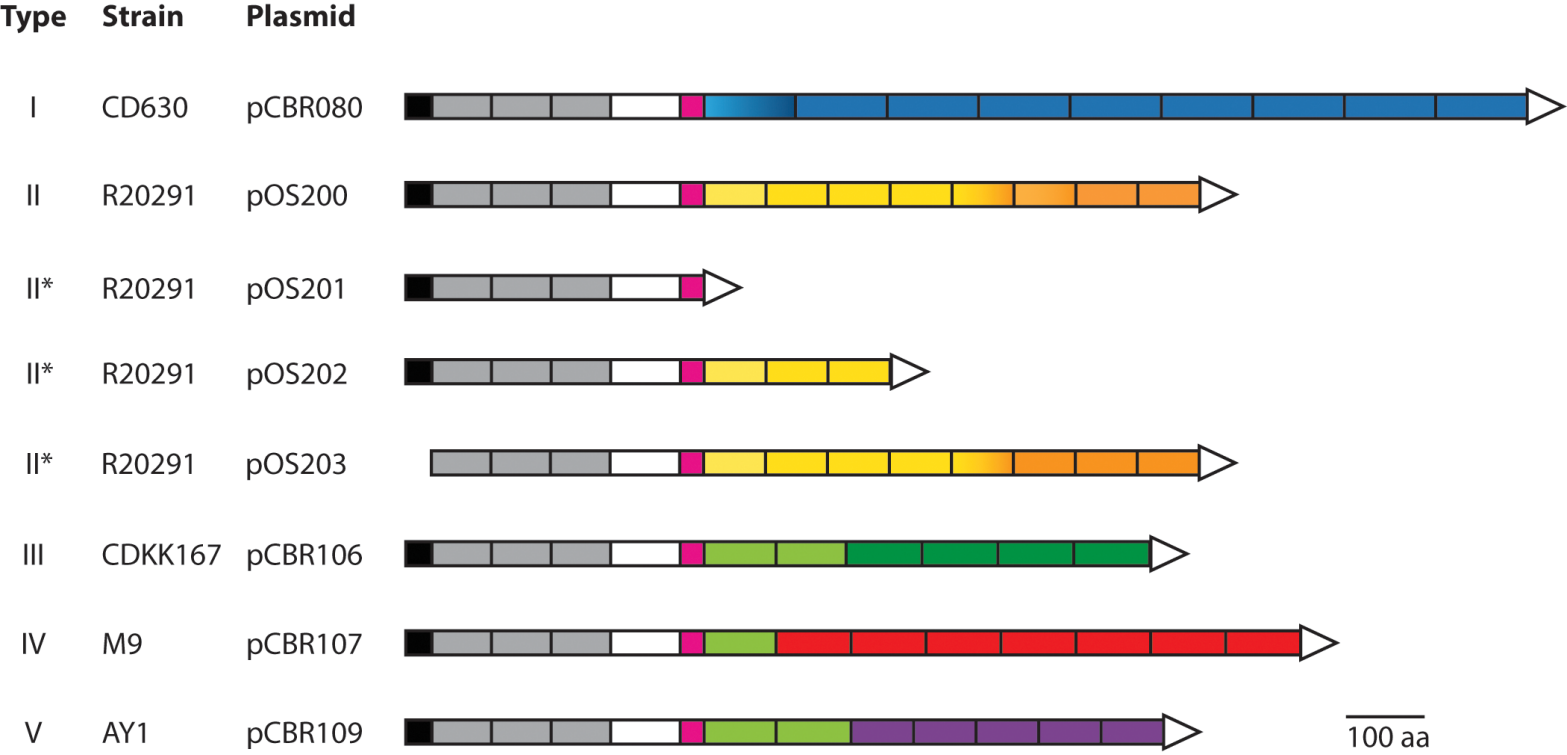
Schematic representation of the CwpV constructions used in this study. The type of CwpV is indicated on the left, along with the strain from which it originates, and the plasmid carrying a copy of the corresponding gene used for expression assays in R20291_OFF_. Color code: Black, signal peptide; gray, cell wall‐anchoring domain (PF04122); white, unknown function; pink, serine/glycine‐rich region; blue, type I repeats; orange, type II repeats; green, type III repeats; red, type IV repeats, purple, type V repeats. Color shades represent different sequence variants of a repeat type. Constructions marked with asterisks are not naturally occurring. Modified from (Reynolds *et al*., 2011).

The CwpV protein is found in all *C. difficile* isolates analyzed to date, and it has been further classified into five different types according to the number and sequence of amino acid repeats composing the variable C‐terminal region (Reynolds *et al*., 2011) (Fig. 3). In order to verify if the antiphage activity observed with the type II CwpV could be extended to other types, we transferred into the R20291_OFF_ strain plasmids carrying one of the other known types, i.e. *cwpV* type I, III, IV and V (Table 1). By incorporating into soft agar overlays (Fortier and Moineau, 2009) dilutions of a phage lysate from ϕCD38‐2, ϕCD111 or ϕCD146 (up to 5 × 10^7^ pfu), we were able to calculate their efficiency of plaquing (EOP) on the different strains, i.e. the proportion of phages that can infect a given strain, compared with a reference strain, in this case wild‐type R20291. As shown in Table 2, we confirmed that all types of CwpV successfully blocked infection by the three siphophages, with EOP values below the maximum phage input used in the assay (i.e. EOP < 5 × 10^−7^) (Table 2). In fact, no phage plaques could be detected with ϕCD38‐2, ϕCD111 or ϕCD146, confirming that all five types of CwpV provided strong antiphage activity against these siphophages.

**Table 2:** Efficiency of plaquing (EOP) for morphologically different phages infecting strains expressing various types of CwpV

CwpV type	*Siphoviridae*			*Myoviridae*	
	ϕCD38‐2	ϕCD111	ϕCD146	ϕMMP01	ϕCD52
I	< 5 × 10^−7^	< 5 × 10^−7^	< 5 × 10^−7^	9 × 10^−3^	1.5 × 10^−2^
II	< 5 × 10^−7^	< 5 × 10^−7^	< 5 × 10^−7^	ND	ND
III	< 5 × 10^−7^	< 5 × 10^−7^	< 5 × 10^−7^	5.6 × 10^−2^	3.3 × 10^−2^
IV	< 5 × 10^−7^	< 5 × 10^−7^	< 5 × 10^−7^	ND	ND
V	< 5 × 10^−7^	< 5 × 10^−7^	< 5 × 10^−7^	9.7 × 10^−2^	1.1 × 10^−1^

ND = not determined.

CwpV is a cell wall–associated protein conserved across the *Clostridium difficile* species. An epigenetic switch controls the expression of the *cwpV* gene in a phase‐variable manner. Bacterial cells that are in the « ON » state express *cwpV* and become resistant to phage infection through blocking of phage DNA injection, in a manner reminiscent of superinfection exclusion systems. CwpV thus represents a novel antiphage system conserved in *C. difficile*.

Next, we wanted to determine if CwpV could also protect *C. difficile* against other morphologically unrelated phages, for example members of the *Myoviridae* family, i.e. phages with non‐flexible contractile tails (Ackermann and Prangishvili, 2012). One limitation that we faced was that only *Siphoviridae* phages from our collection could infect the R20291 strain. To address this, we selected another *C. difficile* strain that was susceptible to infection by myophages and into which conjugation was possible, CD384 (Sekulovic *et al*., 2014). We had two different myophages that could replicate efficiently on this strain, ϕMMP01 (Meessen‐Pinard *et al*., 2012) and ϕCD52 (Fortier and Moineau, 2007). We transferred by conjugation the plasmids carrying the different types of *cpwV* into CD384. Unfortunately, despite several attempts the plasmids carrying type II and type IV *cwpV* could not be transferred. EOP assays with ϕMMP01 and ϕCD52 revealed that CwpV type I was the most effective at preventing infection, especially against ϕMMP01 with an EOP of 9 × 10^−3^, and that type V was the least effective, with an EOP of 1.1 × 10^−1^ with phage ϕCD52 (Table 2). Overall, CwpV seemed to provide some protection against infection by myophages, but the EOP values were at least 3 to 4‐log higher suggesting that CwpV is less effective against this phage family. Taken all together, our results suggest that the antiphage activity provided by all five types of CwpV is stronger with siphophages.

### The C‐terminal domain of CwpV carries the antiphage activity

As the N‐terminal domain of CwpV is involved in cell wall attachment (Willing *et al*., 2015), we hypothesized that the antiphage property was provided by the C‐terminal domain carrying the amino acid repeats. In order to verify this, we constructed two deletion mutants of the CwpV type II protein from R20291. The first plasmid, pOS201, encoded a CwpV‐II lacking the entire C‐terminal domain, and only the N‐terminal domain required for cell wall anchoring was retained (Reynolds *et al*., 2011; Dembek *et al*., 2012) (Fig. 3). The second plasmid, pOS202, lacked the five distal amino acid repeats and retained only the first three proximal repeats within the N‐terminal domain. Both plasmids were transferred by conjugation into R20291_OFF_ and the presence of the protein at the cell surface was verified by immunofluorescence and western blot (Figs S1 and S2). Then, we performed bacterial survival assays to assess the susceptibility to phage infection. The pOS201 plasmid lacking the whole C‐terminal domain did not efficiently protect against ϕCD38‐2 infection, with a survival rate of 33.07% ± 0.72%, which was not significantly different from the rate observed with wild‐type R20291 (20.8% ± 2.5%; Fig. 2). With the pOS202 plasmid encoding a partially deleted CwpV, the protection was intermediate, with a survival rate of 52.07% ± 5.90. These results suggest that the C‐terminal repeats are indeed responsible for the antiphage activity of CwpV.

### CwpV functions as a superinfection exclusion (Sie) system

**Figure 4:**
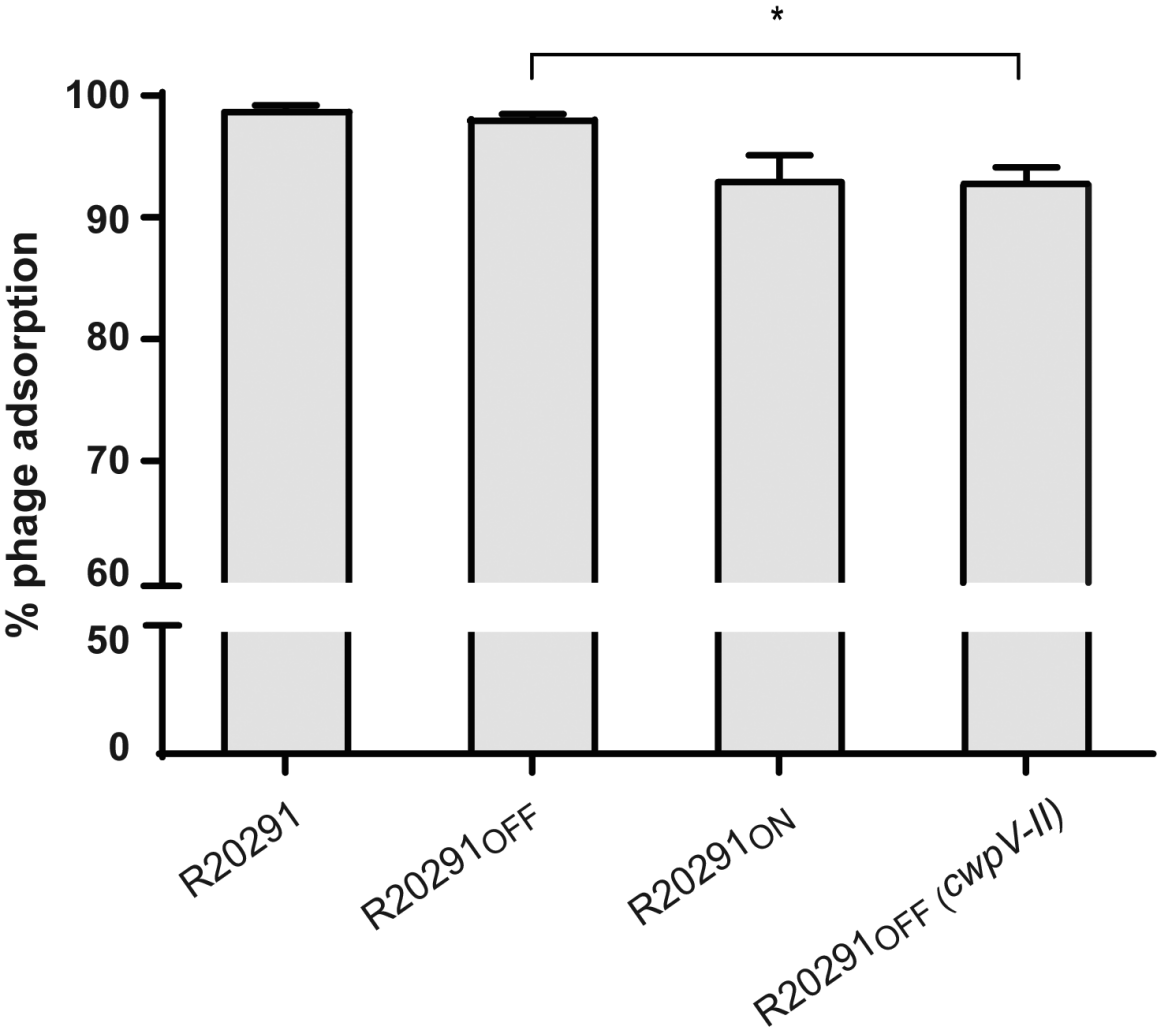
Phage adsorption assay of ϕCD38‐2 on strains expressing or not the type II CwpV. Phages were allowed to adsorb for 30 minute, and then bacteria were pelleted. The adsorption rate is expressed as a percentage of the ratio between non‐adsorbed phages in the supernatant compared to the initial phage inoculum. Vertical bars represent means ± SD of three independent biological replicates, which were also plated in technical triplicates. One‐way ANOVA comparisons were done with R20291_OFF_ as the reference strain (*P < 0.05).

The spot‐test and EOP experiments suggest that when *cwpV* is expressed, no progeny phages are generated (no plaques and no lysis), but the infection process could be abrogated at many different steps. Considering the location of CwpV at the cell surface, the first obvious hypothesis was that CwpV blocked phage adsorption, thus cutting short the whole infection process at the very early steps. To test this, we performed phage adsorption assays with *C. difficile* strains expressing either the full‐length *cwpV‐II* gene from a plasmid [R20291_OFF_(*cwpV‐II*], or from the chromosome (R20291_ON_). We also compared adsorption with a strain that does not express *cwpV* at all (R20291_OFF_). As shown in Fig. 4, results with wild‐type R20291 and the R20291_OFF_ strains were almost identical, with adsorption rates of 98.7% ± 0.5 and 98.0% ± 0.5 respectively. The adsorption on R20291_ON_ and R20291_OFF_(*cwpV‐II*) was similar but slightly lower, with rates of 92.9% ± 2.2 and 92.8% ± 1.4 respectively. Although the difference in phage adsorption between R20291_OFF_ and R20291_OFF_(*cwpV‐II*) was statistically significant (*p* = 0.014), it cannot explain the difference seen in phage infection and the high level of protection observed in spot‐test, EOP and bacterial survival assays. We also performed adsorption tests with phages ϕCD52 and ϕMMP01 on CD384 carrying the empty plasmid pRPF144E, as well as strain CD384 expressing *cwpV‐I*, *cwpV‐III* or *cwpV‐V*. In all cases, adsorption was not significantly affected upon overexpression of *cwpV* (Fig. S3). Altogether, we conclude that the antiphage activity does not result from a defect in phage adsorption but that a step downstream in the infection process is likely affected.

**Figure 5:**
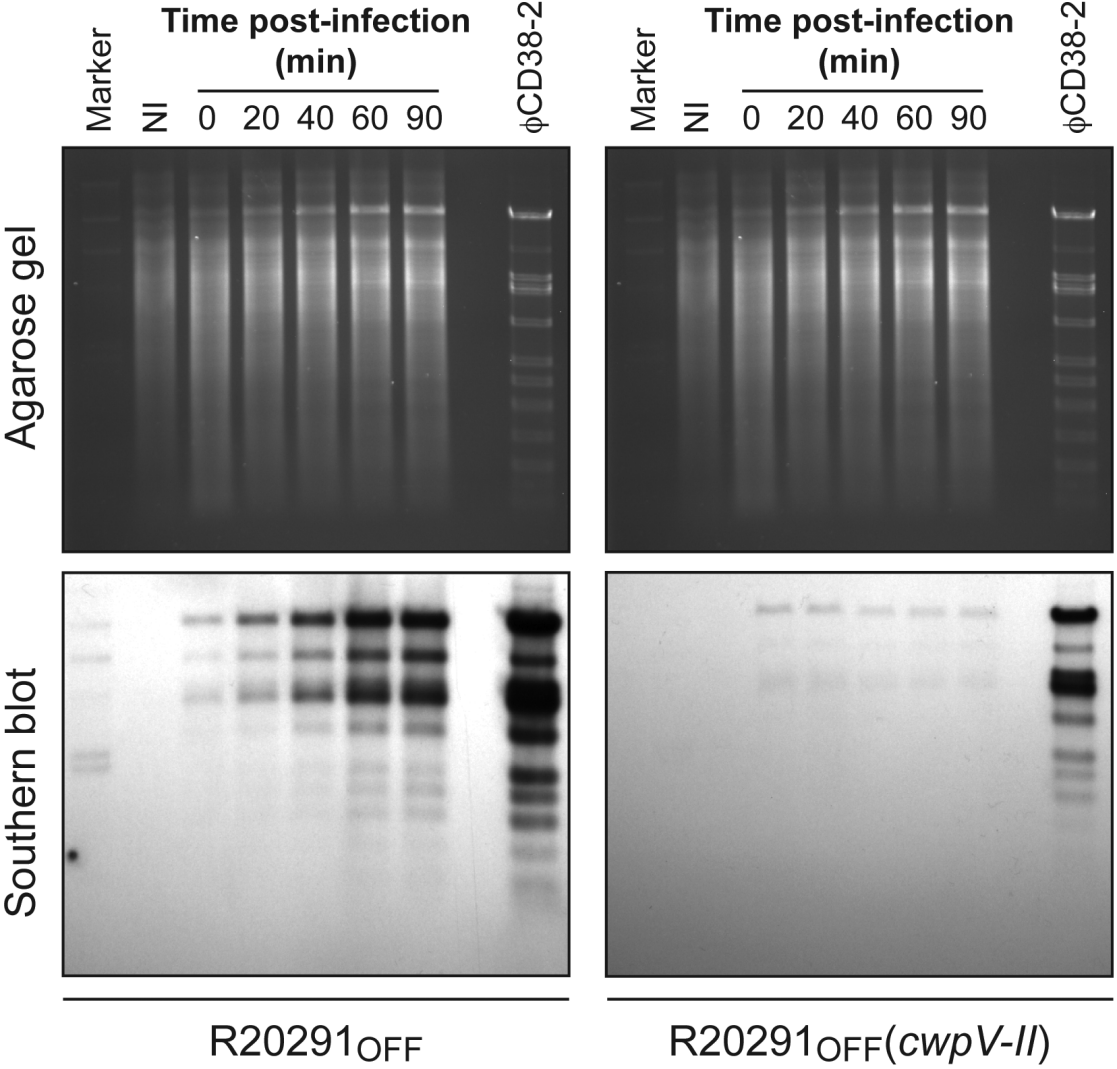
Phage DNA replication assay. The R20291_OFF_ and R20291_OFF_ strain carrying the pOS200 plasmid enabling overexpression of the type II CwpV were each infected with ϕCD38‐2 at a MOI of 1. Samples of the infected cultures were collected at different time points post‐infection, and whole bacterial genomic DNA was extracted. DNA was digested with HindIII and analysed by agarose gel and ethidium bromide staining (upper panel). Southern blot hybridization using a Dig‐labeled whole phage DNA probe was then performed to detect phage DNA replication (lower panel). A non‐infected (NI) control was run in parallel, along with a positive control consisting in the purified ϕCD38‐2 DNA.

A logical hypothesis would be that CwpV blocks phage DNA injection, as the protein is located at the cell surface. To verify this, we performed time‐course phage infection assays during which ϕCD38‐2 genomic DNA replication was monitored by Southern blotting in the absence (R20291_OFF_) or presence [R20291_OFF_(*cwpV‐II*)] of the full‐length *cwpV‐II* gene (Fig. 5). Replication of phage DNA was readily detected after 20 min post‐infection in R20291_OFF_ and reached its maximum intensity after 60–90 minutes, which is in agreement with our previous data on the phage lytic cycle (Sekulovic *et al*., 2011). On the other hand, no phage DNA replication could be detected at any time point in R20291_OFF_(*cwpV‐II*) expressing *cwpV* even after 90 minutes. This result strongly suggests that phage DNA does not enter bacterial cells when CwpV is present at the cell surface. Yet, we could not rule out the possibility that phage DNA was injected, but that intracellular CwpV in transition to the cell surface quickly inhibited phage replication. To address this, we constructed pOS203, a plasmid encoding a CwpV mutant lacking only the signal peptide, to prevent its translocation to the cell surface (Fig. 3). The pOS203 plasmid was transferred into the R20291_OFF_ strain (yielding R20291_OFF_(*cwpV‐II* ΔSigP) to avoid contribution from the chromosomal copy of *cwpV*. The absence of CwpV from the cell surface was confirmed by immunofluorescence on intact cells (Fig. S1) and by analyzing S‐layer extracts by SDS‐PAGE and Western blot (Fig. S2). As expected, CwpV was detected in the cytoplasmic fraction, confirming that the protein was expressed (Fig. S2). Strain R20291_OFF_(*cwpV‐II* ΔSigP) proved to be fully susceptible to infection with phage ϕCD38‐2, with an EOP value of ∼ 1. Therefore, the presence of intracellular CwpV‐II does not interfere with phage DNA replication and the infection proceeds normally. As an additional control experiment, we inactivated the *recV* gene in a strain already carrying ϕCD38‐2, and selected R20291 lysogens with an intact *cwpV* gene under the control of a switch in the ON or OFF configuration. We then monitored prophage induction, with or without mitomycin C treatment and did not observe significant differences in the number of phage particles released (Table S1). Taken together, our results show that CwpV does not interfere with viral DNA replication or early gene transcription when the prophage is already inside the cells. However, CwpV blocks phage DNA injection because phage DNA replication is not detected when exogenous phage particles are used to infect *cwpV*‐expressing cells. Therefore, our data strongly suggest that CwpV functions in a way similar to superinfection exclusion (Sie) systems, although it is not encoded by a prophage.

## Discussion

In this study, we provide experimental evidence showing that the cell wall protein CwpV protects *C. difficile* from phage infection. We show that all five types of CwpV protect against siphophages, whereas types I, III and V protect against infection by myophages, although less efficiently. The C‐terminal, repeat‐containing region of CwpV carries the antiphage activity. Our data further show that phage particles are able to adsorb to their host, but the absence of phage DNA replication suggests that the inhibition occurs at the step of DNA injection. Such antiphage phenotype is reminiscent of the Sie family of proteins, which are generally membrane‐associated prophage‐encoded proteins (McGrath *et al*., 2002; Mahony *et al*., 2008; Labrie *et al*., 2010). CwpV thus represents a novel host‐encoded cell wall–associated and phase‐variable Sie‐like antiphage system ubiquitous within the *C. difficile* species.

Constitutive expression of any type of CwpV from a plasmid was sufficient to completely block infection by three related phages from the *Siphoviridae* family. However, in the R20291_ON_ strain in which *cwpV* is expressed constitutively from its own promoter, protection was somewhat reduced in survival assays with ∼ 75% survival. In addition, EOP values of only 0.5 to 10^−1^ were observed with the R20291_ON_ strain (data not shown), suggesting that the phage‐to‐host ratio is critical for optimal protection. According to a previous study, the amount of CwpV in ‘ON’ cells represents 13.3% of the total surface proteins (Reynolds *et al*., 2011). The major cell wall protein is SlpA that forms a two‐dimensional array into which CwpV is likely inserted. Hence, areas or zones of the cell surface may be devoid of CwpV, which would explain why at high MOI, phages successfully infect the cell. The accumulation of CwpV at the cell surface is greater when expression occurs from a multicopy plasmid. In agreement with this, our qRT‐PCR analyses showed 6.25‐fold more expression in R20291_OFF_(*cwpV‐II*) compared with the R20291_ON_. Likewise, SLP extractions also showed larger amounts of CwpV in extracts from cells expressing the gene from a plasmid compared with locked‐ON cells (Fig. S2). It is also possible that a certain amount of CwpV must be present at the surface to reach a stoichiometric threshold with a bacterial component participating in phage infection. For example, the number of copies of the outer membrane porin OmpC used as a phage receptor by phage T4 is estimated at around 10^2^ to 10^3^ per *Escherichia coli* K‐12 cell (Lu *et al*., 1993). A similar dose‐dependent response has been reported with other antiphage systems such as those from lactic acid bacteria, whereby transferring the antiphage gene onto a high copy plasmid leads to a stronger antiphage phenotype (Bouchard *et al*., 2002; McGrath *et al*., 2002; Sun *et al*., 2006). *C. difficile* cells that turn ‘ON’ the expression of *cwpV* probably produce enough of the protein to acquire significant protection against phage infection in an *in vivo* context, where the MOI for a specific phage is probably low compared with *in vitro* conditions.

We showed that CwpV has a very strong antiphage activity toward all three siphophages tested (ϕCD38‐2, ϕCD111 and ϕCD146), whereas it has only modest activity against myophages (ϕMMP01 and ϕCD52). Of note, the three siphophages used are very similar genetically and morphologically, yet they have slightly different host spectra (Sekulovic *et al*., 2014). On the other hand, the ϕMMP01 and ϕCD52 myophages are different from the siphophages, both genetically and morphologically, and they also have distinct host spectra (Sekulovic *et al*., 2014). The fact that CwpV is active against both phage families suggests that it interferes with a phage structure, host component, or at a step of the infection process common to both groups. One possibility is that both types of phages use different strategies to dock at the membrane interface to inject their DNA and that CwpV interferes less efficiently in the case of the *Myoviridae* phages. Knowledge on the biology and molecular structure of *C. difficile* phages is very limited so it is hard to tell which features of the siphophages and myophages CwpV could target. Likewise, the bacterial receptor(s) recognized by these phages to infect their host is (are) unknown. Such receptor could be a surface carbohydrate, a membrane protein or another surface component (Chapot‐Chartier, 2014). However, adsorption of both *Siphoviridae* and *Myoviridae* phages is not prevented in cells expressing *cwpV*, suggesting that CwpV does not block a phage receptor. Moreover, as we did not detect phage DNA replication inside infected cells, the most logical hypothesis is that CwpV interferes with phage DNA injection. This is further supported by the fact that the infection process was not altered in cells expressing a *cwpV* mutant lacking the signal peptide required to export CwpV to the cell surface and that induction and replication of the ϕCD38‐2 prophage was normal in a lysogenic host expressing *cwpV*. In summary, the interference is likely occurring at the cell surface or membrane interface with the fully processed CwpV. Deleting part or all of the C‐terminal amino acid repeats affected the antiphage activity proportionally, suggesting that the number of repeats rather than their sequence is crucial for CwpV‐mediated antiphage activity.

One of our hypotheses is that CwpV interacts with a structural component of the phage tail, which would be conserved between similar siphophages and that would be sufficiently different or less accessible in myophages. Alternatively, *Myoviridae* and *Siphoviridae* phages might use different host factors and/or mechanisms to inject their DNA, which might involve different types of interactions with the cell membrane and at which point CwpV might interfere. Both *Myoviridae* and *Siphoviridae* phages are part of the order *Caudovirales*, which includes all tailed phages, and it is not uncommon to find phages with identical morphologies, but which are completely different genetically. The structure of phage tails is thus highly conserved, but a main distinction between siphophages and myophages is the presence of a contractile tail sheath surrounding the tail tube of the latter (Fokine and Rossmann, 2014).

Because there is no sequence homology between the siphophages and myophages used in our study, it is hard to tell which phage structural component(s) CwpV could target. One hypothesis is that CwpV interacts with components conserved between the two families and that are less accessible in myophages. The absence of a tail sheath in siphophages leaves the tail tube entirely exposed and might serve as a potential target for CwpV. In myophages such as the coliphage T4, the tail tube becomes exposed only after tail sheath contraction, which is triggered by interactions of phage tail fibers with bacterial host receptors (Leiman *et al*., 2003). In the siphophage λ, the tail tube protein gpV harbors an Ig‐like domain 2 (Big_2) in its C‐terminal region, which protrudes outside of the tail tube, and which was shown to be required for optimal host adsorption and infectivity (Pell *et al*., 2010). The importance of the tail tube and TMP in resistance to superinfection exclusion activity was recently evidenced with coliphages HK97 and HK022 (Cumby *et al*., 2012). HK97 encodes gp15, a membrane protein with superinfection exclusion activity. Although HK97 and HK022 share extensive sequence homology in their capsid and tail tip proteins, HK022 is not susceptible to the action of gp15. However, a HK97/022 hybrid phage in which a genomic region encoding the tail tube and TMP from HK97 has been replaced with that of HK022 is no longer inhibited by gp15. Therefore, gp15 from HK97 prevents phage DNA entry through interaction with the phage tail tube or TMP (Cumby *et al*., 2012).

In Gram‐positive bacteria, only a few examples of Sie systems have been described, including the 142 amino acids lipoprotein Ltp encoded by the *S. thermophilus* phage TP‐J34 (Sun *et al*., 2006). Most phages infecting *S. thermophilus* are related to the lactococcal BK5‐T‐like phages, and Ltp from TP‐J34 confers protection against streptococcal phages (EOP of about 10^−2^) (Sun *et al*., 2006). Surprisingly though, it provides stronger protection against some 936 phages infecting *L. lactis*, in particular phage P008, with an EOP < 10^−9^ (Bebeacua *et al*., 2013). This highlights the potentially wide spectrum of some Sie systems. In a recent study, the crystal structure of the Ltp protein was reported (Bebeacua *et al*., 2013) and mutants of phage P008 capable of bypassing the Ltp antiphage activity were also isolated. Genome sequencing revealed specific mutations in the gene encoding the TMP, suggesting that Ltp targets this specific protein. However, there is no direct experimental evidence of the interaction between Ltp and TMP (Bebeacua *et al*., 2013). In myophages, exposure of the tail tube and TMP is physically and temporally limited contrary to siphophages (Leiman *et al*., 2003). Hence, it is tempting to speculate that CwpV interacts with the tail tube protein or the TMP, which would explain why myophages are less sensitive to the antiphage activity of CwpV. The polymeric and helical nature of the tail tube and TMP (Leiman *et al*., 2003; Plisson *et al*., 2007) could possibly allow for interaction with the repetitive nature of CwpV. In agreement with this, a partially truncated version of CwpV in which five of the distal C‐terminal repeats have been deleted has a reduced antiphage activity against siphophages, whereas complete removal of the C‐terminal repeats cause a complete loss of activity. Despite the use of high phage titers (up to 5 × 10^9^ pfu), we did not detect phage plaques upon infection of a *C. difficile* strain overexpressing *cwpV*, showing that the CwpV antiphage system is very efficient under these conditions. As a result, we were unable to isolate phage mutants capable of overcoming the antiphage activity of CwpV, which could have given us hints about the possible phage target.

Our study was limited by a number of technical hurdles, mainly the availability of a *C. difficile* strain equally susceptible to infection by both siphophages and myophages and that could be genetically manipulated to reintroduce various *cwpV* genes. Indeed, phages infecting *C. difficile* generally have narrow host spectra (Sell *et al*., 1983; Mahony *et al*., 1985; 1991; Dei, 1989; Goh *et al*., 2005; Sekulovic *et al*., 2014), and isolates that are fully susceptible to siphophages are not, or only partially susceptible to infection by myophages and vice versa (Sekulovic *et al*., 2011; 2014; Meessen‐Pinard *et al*., 2012). In addition, the availability of *Siphoviridae* phages to test against CwpV was limited, with only five siphophages described so far (Horgan *et al*., 2010; Sekulovic *et al*., 2011; Hargreaves and Clokie, 2014), three of them being similar (ϕCD38‐2, ϕCD111 and ϕCD146) and another one lacking a propagating host (ϕCD24‐1; our unpublished data).

### Concluding remarks

Phase variation in the bacterial world has often been associated with immune evasion during pathogenic infections. But in fact, phase variation might serve multiple other functions associated with virulence, persistence and colonization (Li *et al*., 2002; Kearns and Losick, 2005; van der Woude, 2006; Tauseef *et al*., 2013; Alamro *et al*., 2014). For example, phase‐variable antiphage systems can protect subpopulations from lytic phage attacks (van der Woude, 2006; Hoskisson and Smith, 2007), without compromising the capacity of the whole bacterial population to acquire new genetic material by horizontal transfer (Brussow *et al*., 2004; Fortier and Sekulovic, 2013). The expression of a phase‐variable antiphage system such as CwpV thus becomes highly relevant in a context where multiple phage marauders are present in the mammalian gut (Mills Susan *et al*., 2013). Further investigations will be required to determine the importance of CwpV in antiphage protection during gut colonization *in vivo*.

## Experimental procedures

### Bacterial strains, bacteriophages and plasmids

A complete list of bacterial strains, plasmids and bacteriophages used in this study is presented in Table 1. *C. difficile* was routinely grown inside an anaerobic chamber (Coy Laboratories), under anaerobic atmosphere (10% H_2_, 5% CO_2_ and 85% N_2_) at 37°C in pre‐reduced brain hearth infusion (BHI) broth or TY broth (3% tryptose, 2% yeast extract, pH 7.4). Thiamphenicol (15 μg ml^−1^) and norfloxacin (12 μg ml) were added when necessary. *E. coli* was grown aerobically in Luria–Bertani broth in a shaking incubator at 37°C with appropriate antibiotics (chloramphenicol 25 μg ml^−1^ or kanamycin 50 μg ml^−1^) when necessary. Concentrated phage lysates (≥ 10^9^ pfu ml^−1^) were prepared by standard phage induction and amplification protocols as described elsewhere (Sekulovic *et al*., 2011) and stored at 4°C.

### Determination of phage titers and efficiency of plaquing (EOP)

For determination of phage titers, we used a standard soft agar overlay method (Fortier and Moineau, 2009) with 0.5 ml of a log‐phase sensitive strain and 10 mM CaCl_2_ + 0.4M MgCl_2_. For rapid evaluation of bacterial sensitivity to phage infection, 5 μl of serially diluted phage lysates were spotted directly on top of the soft agar overlay. Clear zones of lysis in the bacterial lawn were indicative of a productive phage infection. The EOP was used for quantitative analysis of bacterial sensitivity to phage infection and consisted in dividing the phage titer (in plaque forming units (pfu) ml^−1^) of a given phage on the test strain, by the phage titer (in pfu ml^−1^) of that phage on a sensitive reference strain (Fortier *et al*., 2005).

### Isolation of R20291_OFF_ and R20291_ON_ clones

Inactivation of the bacterial recombinase RecV from strain R20291 (CDR20291_1004) using the ClosTron system has been described previously (Reynolds *et al*., 2011; Sekulovic and Fortier, 2015). Colony PCR on putative *recV* mutants was performed in order to identify clones for which the genetic switch controlling the expression of the *cwpV* gene (Emerson *et al*., 2009) was locked either in the ‘ON’ (primer pair LCF 801 + LCF 714) or ‘OFF’ (primer pair LCF 796 + LCF 797) configuration (Table S1). To create lysogens with the ‘ON’ or ‘OFF’ configuration, the ϕCD38‐2 prophage was introduced first into strain R20291 to create a lysogen, and then a plasmid carrying the ClosTron construction was introduced by conjugation to inactivate the *recV* gene. As both ‘ON’ and ‘OFF’ configurations exist in a lysogenic population, we screened colonies to isolate an R20291_ON_lysogen and R20291_OFF_lysogen. Surface protein extracts from positive clones were analyzed on polyacrylamide gel electrophoresis (SDS‐PAGE) followed by Coomassie blue staining to validate the presence (‘ON’ clones) or the absence (‘OFF’ clones) of CwpV protein at the cell surface.

### Cloning and expression of CwpV‐related constructions

The full‐length *cwpV‐II* gene including the putative ribosome‐binding site was amplified by PCR from *C. difficile* R20291 using primers LCF 756 and LCF 757. Truncated versions of the *cwpV‐II* gene were also amplified by PCR as follows: primers LCF756 and LCF896 were used to amplify the region encoding the N‐terminal domain of CwpV‐II, whereas primers LCF 756 and LCF 897 were used to amplify the region spanning the N‐terminal domain + 3 proximal amino acid repeats. The PCR products were cloned in place of the *gusA* gene downstream of the constitutive *P_cwp2_* promoter in the pRPF144 plasmid (Fagan and Fairweather, 2011) (Table 1) using SacI and BamHI restriction enzymes. The resulting plasmids were named pOS200, pOS201 and pOS202 respectively. An in‐frame deletion of the signal peptide from CwpV was also constructed as follows. The signal peptide was identified using SignalP 4.1 (http://www.cbs.dtu.dk/services/SignalP/) (Petersen *et al*., 2011), and further verified manually based on conserved characteristics for Gram‐positive species (von Heijne and Abrahmsén, 1989; van Roosmalen *et al*., 2004). Next, the 5′ fragment of the *cwpV* gene including the ribosome binding site and ATG start codon was PCR‐amplified with primers LCF 941 and LCF 942. A 3′ fragment downstream of the signal peptide and including the N‐terminal cell wall binding domain, C‐terminal repeats and a putative transcriptional terminator was PCR‐amplified with primers LCF 943 and LCF 944. A Gibson isothermal assembly procedure (Gibson *et al*., 2009) was then used to clone the two PCR fragments into a modified pRPF144 backbone. Briefly, the pRPF144 plasmid was digested with SacI and BamHI to remove the *gusA* gene. The remaining plasmid fragment was then treated with T4 DNA polymerase (NEB) to generate blunt ends, and ligated with T4 DNA ligase (NEB) to give pRPF144E. The plasmid was linearized with BamHI and purified by EZ‐10 spin column DNA cleanup kit (BioBasic). Equimolar ratios of 5′ and 3′ *cwpV* fragments and linearized pRPF144E were pooled in a total volume of 5 μl and mixed with 15 μl of Gibson enzyme–reagent master mix containing 5% PEG‐8000, 100 mM Tris–HCl pH 7.5, 10 mM MgCl_2_, 10 mM DTT, 200 nM each of the four dNTPs, 1 mM NAD, 0.08 U T5 exo (Epicentre), 80 U *Taq* ligase and 0.5 U Phusion polymerase (NEB). The reaction mix was incubated at 50°C for 1 h, and 1 μl was used to transform *E. coli* CA434 competent cells using standard procedures (Sambrook and Russell, 2001). Positive clones carrying the correct constructs were verified by DNA sequencing and subsequently transferred by conjugation into *C. difficile* R20291_OFF_ as described previously (Sekulovic and Fortier, 2015).

### Immunoblotting for detection of CwpV

Expression of the recombinant proteins was confirmed by Western immunoblotting. Briefly, *C. difficile* cell surface proteins were glycine‐extracted as previously described (Sekulovic and Fortier, 2015). Following S‐layer extraction, cells were washed in 1× PBS and then mechanically lysed using 0.5 g of acid‐washed glass beads (106 μm, Sigma) in a FastPrep apparatus (MP Bioscience) for 45 second at 4 m s^−1^. Proteins were separated by 10% SDS‐PAGE and then transferred on nitrocellulose membranes using standard procedures (Sambrook and Russell, 2001). Immunoblotting was performed either with chicken IgY primary antibodies specific to the N‐terminal part of CwpV protein (Cat. No. ABIN2039021; Immune Biosolutions, Sherbrooke, QC) or rabbit primary antibodies raised against the first two C‐terminal repeats (Reynolds *et al*., 2011). Primary antibodies were detected using either an HRP‐conjugated goat anti‐rabbit secondary antibody (Life Technologies) or an HRP‐conjugated alpaga anti‐chicken secondary antibody (Immune Biosolutions) following manufacturers' recommendations.

### Bacterial survival assays

Bacterial survival assays were performed as follows. *C. difficile* overnight cultures were used to inoculate 5 ml of fresh TY broth and bacteria were grown until exponential phase (OD_600nm_ = 0.5). Then, 0.9 ml of the culture was taken and mixed with ϕCD38‐2 phage lysate in order to obtain a MOI of 1. MgCl_2_ and CaCl_2_ were added to a final concentration of 10 mM each, and the volume was completed to 1 ml with sterile TY broth. An uninfected control without phage was run in parallel. Both samples were mixed by inversion and incubated for 15 minutes at 37°C, after which aliquots were quickly diluted in triplicate in 96 well plates. Then, 0.1 ml of these dilutions was plated on BHI agar and incubated overnight. The next day, colonies were counted, and the ratio between infected and uninfected controls was indicative of bacterial sensitivity to phage infection and expressed as percentage of survival ([infected/uninfected] × 100).

### Phage adsorption assays

Phage adsorption assays were performed as previously described with slight modifications (Hyman and Abedon, 2009). Briefly, bacteria from an overnight culture were inoculated in TY broth and grown until exponential phase (OD_600nm_ = 0.5). Then, 0.9 ml of cells was mixed with 1 × 10^4^ pfu in the presence of salts (10 mM CaCl_2_ and MgCl_2_), and the volume was completed to 1 ml with fresh TY broth. Phages were allowed to adsorb for 30 minutes at 37°C after which cells were collected by centrifugation. Free phages in the supernatant that did not adsorb were counted on standard soft agar overlays and titers were compared with the initial phage inoculum. The percentage of adsorption was calculated with the following formula: 100 – ([residual titer/initial titer] × 100).

### Detection of phage DNA replication

Phage DNA replication within bacterial cells was monitored as previously described with modifications (Domingues *et al*., 2004). Briefly, bacteria from an overnight culture were inoculated in 100 ml of fresh TY broth and allowed to grow until exponential phase (OD_600nm_ = 0.5) at which point CaCl_2_ and MgCl_2_ were added directly to the culture at final concentrations of 10 mM each. One 5 ml aliquot was immediately taken as a non‐infected control. Then, the phage lysate was added at a MOI of 1 and 5 ml aliquots were taken at 0, 20, 40, 60 and 90 minutes. Each sample was mixed with an equal amount of cold acetone‐ethanol mix (1:1) in order to stop the replication machinery in the cell and stabilize the DNA. Cells were harvested by centrifugation, washed in 1× PBS and total DNA was extracted by phenol‐chloroform extraction followed by ethanol precipitation. Two micrograms of total DNA was digested with HindIII (NEB) following manufacturer's instructions and run through a 0.8% agarose gel. DNA transfer and Southern hybridization with whole‐phage digoxigenin (DIG)‐labeled probe were performed as described previously (Fortier and Moineau, 2007).
