# Supplementary material for: The C lostridium difficile cell wall protein CwpV confers phase‐variable phage resistance
Source: Mol Microbiol. 2015 Aug 8;98(2):329–42. doi: 10.1111/mmi.13121 (PMC4737114; doi:10.1111/mmi.13121)
Supplement: Supplementary file 1 [file MMI-98-329-s001.pdf]

Supplementary information for:

The *Clostridium difficile* Cell Wall Protein CwpV Confers Phase-Variable Phage Resistance

Ognjen Sekulovic[1], Maicol Ospina Bedoya[1], Amanda S Fivian-Hughes[2§], Neil Fairweather[2] and Louis-Charles Fortier[1*]

[1] Département de microbiologie et d'infectiologie, Faculté de médecine et des sciences de la santé, Université de Sherbrooke, Québec, Canada

[2] Centre for Molecular Bacteriology and Infection, Department of Life Sciences, Imperial College London, London, UK

[*]For correspondence: E-mail: Louis-Charles.Fortier@USherbrooke.ca; Tel. (819) 821-8000 x 75322; Fax (819) 820-6831

Running title: CwpV confers phage resistance in *C. difficile*

Keywords: *Clostridium difficile*, bacteriophage, phage resistance, antiphage system, Cell wall protein

§ Current address: Synthace Ltd., The London Bioscience Innovation Centre, 2 Royal College Street, London, NW1 0NH, UK

Fig. S1. Immunofluorescence detection of CwpV expression. The presence of CwpV at the bacterial surface was assessed by immunofluorescence using antibodies directed against the C-terminal repeats of the protein. We can notice the absence of the protein in the "OFF" strain that does not express the endogenous copy of the *cwpV* gene ($R20291_{OFF}$), in the strain expressing the truncated version of the protein lacking all the C-terminal repeats ($R20291_{OFF}$(*cwpV-II* 3reps)), and in the strain with a CwpV lacking the signal peptide ($R20291_{OFF}$(*cwpV-II* ΔSigP)).

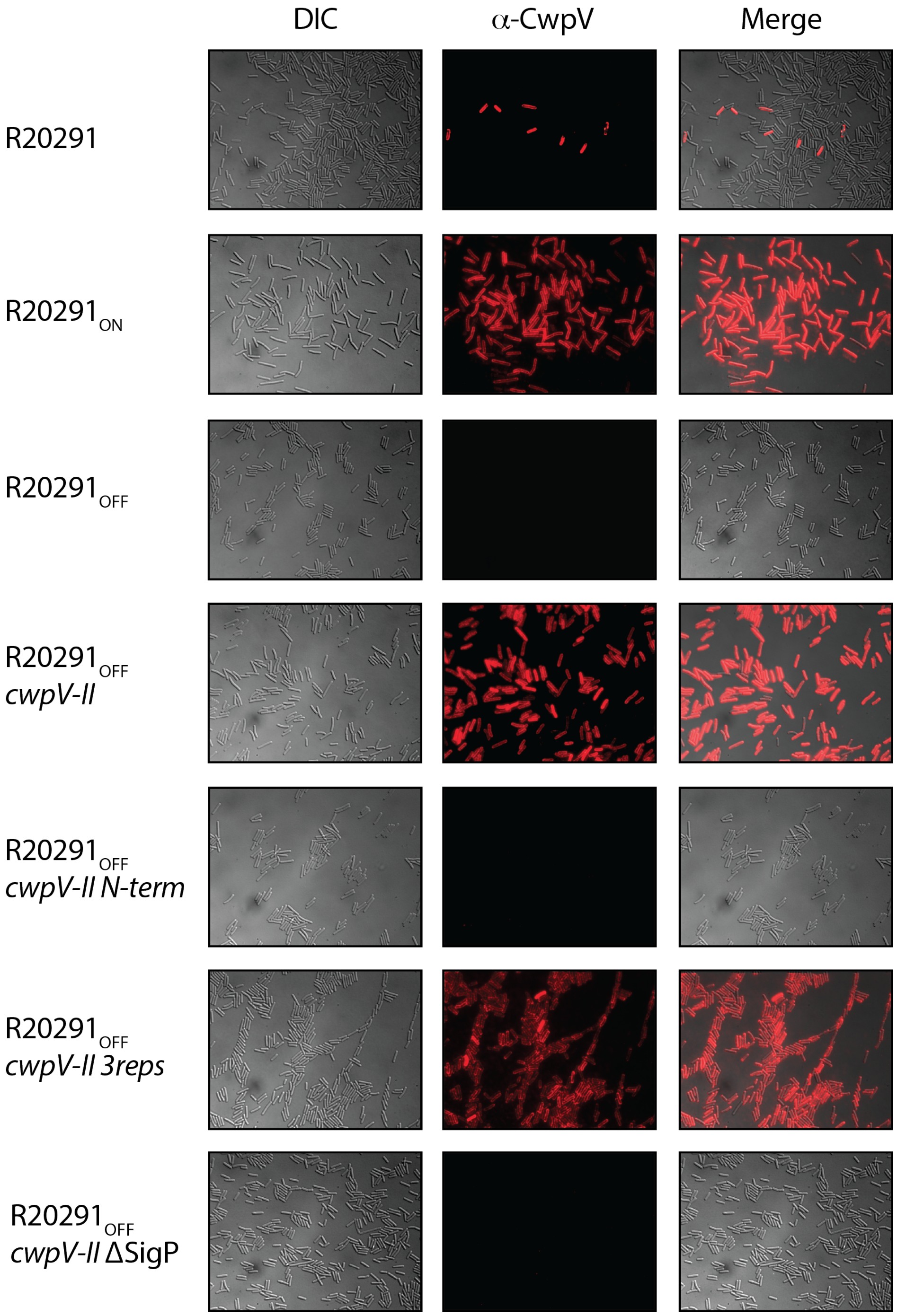

Fig. S2. Western immunoblotting for detection of various CwpV constructs in *C. difficile* surface-layer extracts. A) Coomassie Blue staining (upper panel) and Western immunoblot detection (lower panel) of CwpV-II constructions in S-layer extracts of R20291 (A) or CwpV-I, III and V in S-layer extracts from strain CD384. An anti-CwpVNter primary antibody targeting the N-terminal portion of the protein was used. The presence of a weak band in CD384 is due to the expression of the endogenous *cwpV* gene, which is not expressed in the R20291$_{OFF}$ (*recV* locked "OFF" strain). C) Detection of CwpV-II in S-layer extracts (SLPs) and in the cytosol (Cyt.) using anti-CwpVrptI primary antibodies targeting the C-terminal repeats. The dotted line arrows indicate the N-term fragment and the solid line arrows indicate the C-term fragment. The pOS203 plasmid expresses a version of CwpV lacking the signal peptide, resulting in the accumulation of CwpV in the cytosolic fraction only.

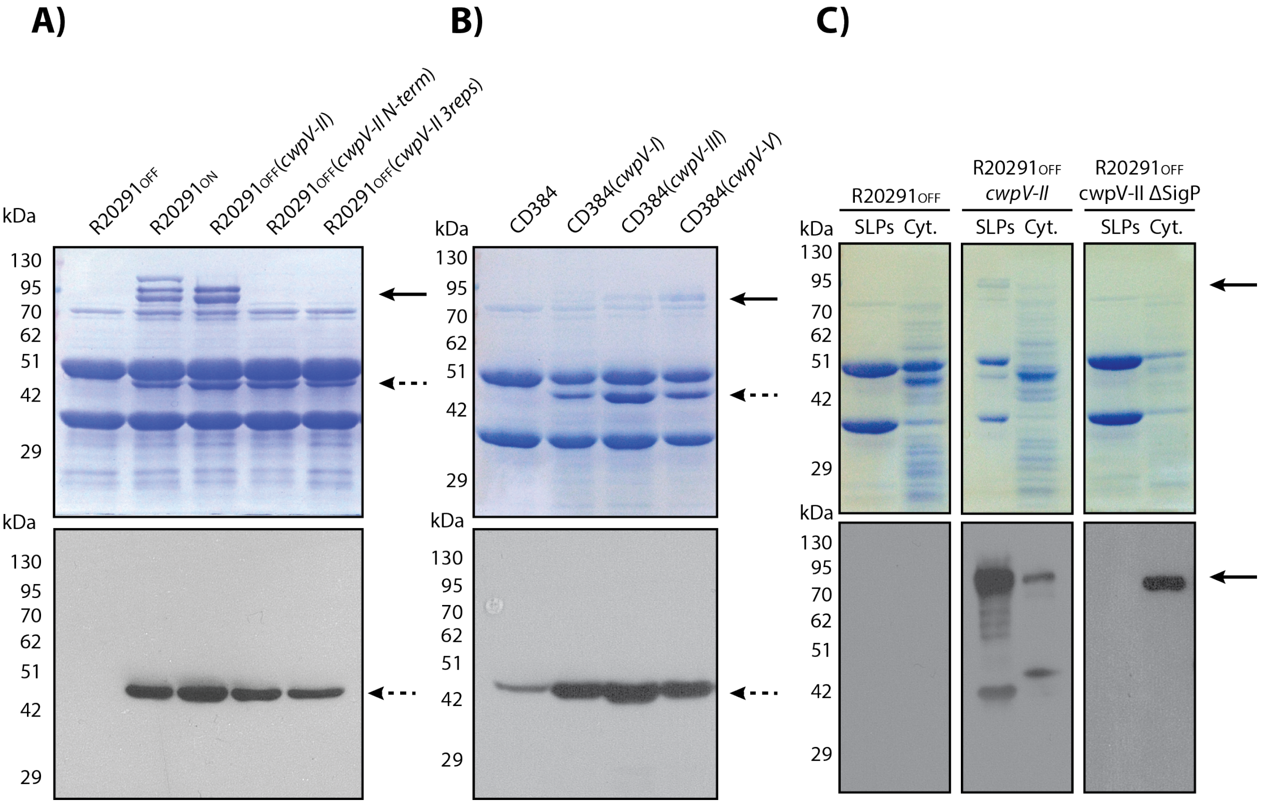

Fig. S3. Phage adsorption assay with ϕCD52 and ϕMMP01 on strains expressing or not the CwpV type I, III or V. Phages were allowed to adsorb for 30 min and then bacteria were pelleted. The adsorption rate is expressed as a percentage of the ratio between non-adsorbed phages in the supernatant compared to the initial phage inoculum. Horizontal bars represent means of three independent biological replicates (symbols), which were also plated in technical triplicates. One-way ANOVA comparisons were done with CD384 + pRPF144E as the reference strain but the differences did not reach statistical significance ($p < 0.05$).

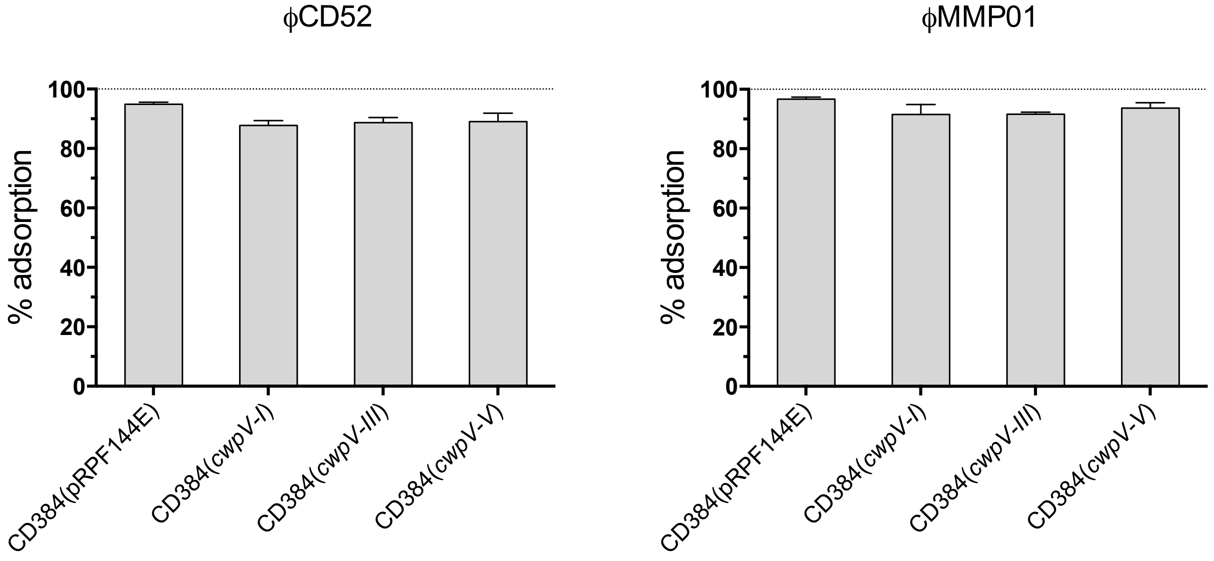

Table S1. List of primers used in this study.

Primer	Target	Strand	Sequence (5'- 3')	Product size (bp)
LCF 312	ϕCD38-2 detection	Fwd	AGCGGTATCGGCTTGGTTGTAGAT	537
LCF 313		Rev	TGCTAGTTTCCTGTCAAGGTCGCT	
LCF 796	*cwpV* OFF switch	Fwd	CGCAATTATTTGTTTTTCATATGGATAAAATTGG	163
LCF 797		Rev	GATTTTTATGTTAATGAATTGTTATAAAAAACATGG	
LCF 801	*cwpV* ON switch	Fwd	GGTAAGTTTGATTTTTATGTTAATGAATTG	223
LCF 714		Rev	CAGTTTGTGCACTAGCTATGCCTGC	
LCF 756	*cwpV-II*	Fwd	NNNNGAGCTCGTATCCTTTAGAATTAGAACGGGAAC	3704
LCF 757		Rev	NNNNGGATCCCTTTACATGATAAAAAGGCTGTG	
LCF 896	*cwpV-II N-terminal*	Rev	NNNNGGATCCTTTATATTCACCTACATTTGTTCCCTC	1743
LCF 897	*cwpV-II 3 repeats*	Rev	NNNNGGATCCAGCATATTCTCCCTCTGCTGTTCC	2454
LCF 941	Gibson 5' *cwpV* fragment	Fwd	AAATAAGGAAAAAATAATAAGAACAATTCATTAACATAAAAATCAAAC	291
LCF 942		Rev	CATTTTATTTTCTTCCCCCTCATTTTATTTTCTTCCCCCTTG	
LCF 943	Gibson 3' *cwpV* fragment	Fwd	AGGGGGAAGAAAATAAAATGCAAACTGTGGCAACAAATTTAAC	3567
LCF 944		Rev	ACTGGCGGCCGTTACTAGTGCTTTACATGATAAAAAGGCTGTG	

Table S2. Prophage induction (with or without mitomycin C treatment) of R20291 lysogens expressing or not *cwpV-II*

	Prophage induction (pfu/mL)	
	**Spontaneous**	**Mitomycin C**
$R20291_{OFF}$ lysogen	$3.6 \pm 2.2 \times 10^5$	$8.5 \pm 8.5 \times 10^7$
$R20291_{ON}$ lysogen	$2.8 \pm 1.1 \times 10^6$	$4.1 \pm 3.6 \times 10^7$
